# Imprint of Initial Education and Loss of Ly49C/I in Activated Natural Killer Cells of TAP1-KO and C57BL/6 Wildtype Mice

**DOI:** 10.3389/fimmu.2022.818015

**Published:** 2022-07-14

**Authors:** Neha D. Patil, Olivia Domingues, Cécile Masquelier, Maud Theresine, Oceane Schlienger, Clinton Njinju Amin Asaba, Marine Thomas, Carole Seguin-Devaux, Hortense Slevogt, Markus Ollert, Jacques Zimmer

**Affiliations:** ^1^ Department of Infection and Immunity, Luxembourg Institute of Health (LIH), Esch-sur-Alzette, Luxembourg; ^2^ Doctoral School in Systems and Molecular Biomedicine, University of Luxembourg, Esch-sur-Alzette, Luxembourg; ^3^ Centre for Innovation Competence (ZIK) Septomics, Host Septomics, Jena University Hospital, Jena, Germany; ^4^ Department of Dermatology and Allergy Center, Odense Research Center for Anaphylaxis, University of Southern Denmark, Odense, Denmark

**Keywords:** natural killer cells, NK cells, education, major histocompatibility complex class I, inhibitory receptors, TAP-KO

## Abstract

Natural killer (NK) cells are important effectors of the innate immune system and participate in the first line of defense against infections and tumors. Prior to being functional, these lymphocytes must be educated or licensed through interactions of their major histocompatibility complex class I molecules with self-specific inhibitory receptors that recognize them. In the absence of such contacts, caused by either the lack of expression of the inhibitory receptors or a very low level of major histocompatibility complex class I (MHC class I) proteins, NK cells are hypo-reactive at baseline (*ex vivo*). After stimulation (assessed through plate-bound antibodies against activating receptors or culture in the presence of cytokines such as interleukin (IL)-2 or IL-15) however, they can become cytotoxic and produce cytokines. This is particularly the case in transporter associated with antigen processing (TAP)-deficient mice, which we investigated in the present study. Transporter associated with antigen processing transports endogenous peptides from the cytosol to the endoplasmic reticulum, where they are loaded on nascent MHC class I molecules, which then become stable and expressed at the cell surface. Consequently, TAP-KO mice have very low levels of MHC class I expression. We present a study about phenotypic and functional aspects of NK cells in two mouse strains, C57BL/6 wildtype and TAP1-KO in spleen and lung. We observed that in both types of mice, on the same genetic background, the initial pattern of education, conferred to the cells *via* the inhibitory receptors Ly49C/I and NKG2A, was maintained even after a strong stimulation by the cytokines interleukin-2, interleukin-12, interleukin-15 and interleukin-18. Furthermore, the percentages of activated NK cells expressing Ly49C/I and Ly49I were strongly down-modulated under these conditions. We completed our investigations with phenotypic studies of NK cells from these mice.

## Introduction

Natural Killer (NK) cells are the founding members of the innate lymphoid cell (ILC) family comprising in addition several other populations called innate lymphoid cells types 1, 2 and 3, as well as lymphoid tissue inducer cells (1). All the latter are predominantly cytokine producers, whereas NK cells are likewise able to release cytokines and chemokines but are also endowed with cytotoxic properties, further subdivided into natural cytotoxicity (killing of targets without prior sensitization or immunization) and antibody-dependent cellular cytotoxicity (ADCC), which occurs when the Fc portion of antibodies bound to a target cell interacts with the activating NK cell receptor CD16 (FcγRIIIa) (2, 3). Natural Killer cells are extensively studied in human and mouse for their anti-tumoral and anti-infectious properties that are therapeutically exploitable and constitute a true hope for the future of immunotherapy (4, 5). Since the first descriptions of NK cells in the mid-seventies, their capacity to kill tumor cells and viral-infected cells has been observed in many *in vitro* and *in vivo* investigations. Upon recognition of a target cell, the granule content of the cytolytic vesicles (perforin, granzymes, and, in human but not in the mouse, granulysin) is released and induces apoptosis of the abnormal cell (6).

Before reaching this step, NK cells integrate signals from their activating and inhibitory receptors, respectively (AR and IR), and the target cell is eliminated when the activating messages predominate (7). The best studied IR are specific for major histocompatibility complex (MHC) class I molecules, and a normal level of the latter is characteristic for healthy cells that are consequently identified as such and spared by the NK cells. Their absence is recognized as “missing self” and induces target cell lysis in the presence of sufficient activating messages (7–9). Besides the concept of the balance between signals transmitted through AR and IR (7), another important factor governing NK cell functions is education or licensing (10–12). These terms designate the interaction of IR with autologous MHC class I molecules during NK cell development, which is necessary for the cell to become functional (10–12), although non-MHC class I ligands can educate NK cells *via* different IR (11). Therefore, NK cell education relies on the paradox that they only become active after having received a signal through an IR that later can inhibit NK cell functions. In the absence of this phenomenon, due either to the lack of expression of one or several self-specific IR on a NK cell or to a general MHC class I deficiency of the cellular environment, such as observed in beta-2 microglobulin (β2m) and/or transporter associated with antigen processing (TAP) defects, NK cells remain uneducated (unlicensed) and hypo-reactive. This observation was made both in human (13–15) and in knockout mouse strains, such as β2m-KO, TAP1-KO, β2m/TAP1 double KO (16, 17): *ex vivo* NK cells display low or absent cytotoxicity and low cytokine production, but become functionally very active upon cytokine-mediated stimulation and then kill autologous Con A T cell blasts (16). In human TAP deficiency, activated NK cells kill autologous B lymphoblastoid cells (Epstein-Barr virus-transformed immortalized B lymphocytes) and skin fibroblasts, whereas they surprisingly spare self T-PHA blasts (18–20).

Natural killer cell education is in the focus of interest of several groups. Different models have been elaborated, such as the arming model (21), the disarming model (21, 22), the rheostat model (23, 24), the *cis*-*trans* model (25, 26) and the confinement model (11, 12). More recently, it has been shown that TRP Calcium channels dynamically regulate NK cell licensing on the level of the content of cytolytic proteins in the secretory lysosomes (that degranulate upon target cell recognition) (27). Moreover, and in accordance with the *cis-trans* interaction model, the role of NK cell-intrinsic MHC class I molecules for tuning has been emphasized (28).

All these different concepts have mostly been established with short-term activated NK cells, for example taken *ex vivo* the day after poly (I:C) or tilorone administration. In the present work, we wanted to rather explore what happens in mouse NK cells in terms of the production of their major cytokine, namely interferon gamma (IFNγ), after culture with interleukin 2 (IL-2) for five days and then an overnight re-stimulation with IL-2 alone, (IL-2, IL-12, IL-15) and (IL-2, IL-12, IL-18), respectively. The first condition was the negative control, as in the mouse IL-2 alone does not induce significant IFNγ production. In contrast, the last cytokine combination represented the positive control, as IL-12 and IL-18 together activate the production of a maximal amount of IFNγ. Finally, the IL-12 plus IL-15 association was supposed to lead to an intermediate level of stimulation, for which differences between NK cell subsets, defined by the presence or absence of self-specific IR, could be visible.

We choose to work with the most frequently used mouse model in immunology, the C57BL/6 (B6) strain, and its TAP1-KO littermates. In B6 mice, the self MHC class I-specific IR are Ly49C/I, recognizing the classical MHC class I molecules H-2K^b^ (and H-2D^b^), and CD94/NKG2A (NKG2A), binding to the non-polymorphic molecule Qa-1^b^, the mouse equivalent of HLA-E (29, 30). Consequently, NK cells expressing either one or both IR are supposed to be educated, whereas their double negative (Ly49C/I-NKG2A-) counterparts are expected to be hypo-reactive. In contrast, in TAP1-KO mice, the entire NK cell population should in principle be functionally deficient. The reason for the choice of TAP1-KO animals was to undertake an *in vitro* study of their NK cells before switching to a mouse *in vivo* model of a chronic bacterial infection of the airways in order to detect what goes wrong in this context. Transporter associated with antigen processing-deficient human patients suffer severely from such pathologies, which usually evolve to bronchiectasis (13, 14). Understanding the pathophysiology of this phenomenon and the potential link between NK cells and bacterial infections might be useful even for patients without a MHC class I deficiency.

An additional layer of complexity in NK cell education stems from the observation that Ly49C interacts with its ligand H-2K^b^ in *cis*, on the same NK cell membrane, and simultaneously in *trans*, with the same ligand on surrounding target cells, which leads to an optimal calibration of NK cell reactivity even in the presence of only a minor down-regulation of the MHC class I molecule in the cellular environment. This phenomenon had initially been demonstrated by the Held group for the receptor-ligand pair Ly49A – H-D^d^ (26). As the 5E6 monoclonal antibody binds to both the IR Ly49C and Ly49I, the former might actually not been well recognized by the antibody due to its *cis* interaction, which could render the epitope inaccessible. We unsuccessfully tried to obtain aliquots of the non-commercially available Ly49C-specific antibody clone 4LO3311 (31) from several groups, and attempted acid stripping to disrupt the *cis* interaction. By staining Ly49I with the specific antibody YLI-90, the problem was resolved at least indirectly, by showing the “educational” effect of Ly49C compared with no such effect for Ly49I.

In summary, our investigations show that the initial pattern of NK cell education leaves its imprint even after six days of intense cytokine-mediated stimulation in B6 animals, and, surprisingly, that there is an unequal functional distribution between NK cell subsets of activated TAP-KO littermates, largely matching the one observed in B6 mice. Overall, we confirm that a defective TAP results in somewhat reduced NK cell functions *ex vivo*, but that the cells display the same educational profile than their wildtype counterparts after a strong cytokine-mediated stimulation, which should in principle not be the case. The IR Ly49I is well expressed on NK cells from both strains, *ex vivo* and after activation, but does not really seem to play a major role in NK cell education. Furthermore, we were able to detect Ly49C from a functional point of view (acting as an educating IR), but not with absolute certainty as a reliable phenotypic NK cell marker.

## Material and Methods

### Ethical Statement

The animal studies were approved by the Animal Welfare Structure (AWS) and the experiments were carried out in accordance with the European Union directive 2010/63/EU as incorporated in Luxembourgish law for the care and use of laboratory animals. The official authorization for our protocol was given by the Luxembourgish Ministry of Health under the number DII-2017-02.

### Mice

Wildtype (TAP1+/TAP1+) and TAP1-KO (TAP1-/TAP1-) littermates on the C57BL/6 (H-2b) genetic background, obtained through intercross of heterozygous F1 (TAP1+/TAP1-) mice aged 8-12 weeks, were used. The genetic characteristics (homozygous wildtype, heterozygous, and homozygous TAP1-) were established through genotyping. Mice were bred and maintained at the Luxembourg Institute of Health’s specific pathogen-free animal facility. They were fed a standard maintenance chow and followed a 12-h light dark cycle at 22-23°C and 45-65% relative humidity.

### Cell Preparation

Lungs and spleens were extracted from WT and TAP1-KO mice. Lungs were incubated in 1.6 ml digestion solution (for 15 ml, 20 mg collagenase II, 10% FBS, 750 U benzonase, 15 µl of 1M MgCl_2_, PBS) for one hour at 37°C. Spleens and the digested lungs were passed through a 40 µm cell strainer (Corning) with the back of a syringe plunger to make a single cell suspension. Red blood cell lysis was performed by ACK lysing buffer (Gibco™). Murine T-lymphoma cell lines YAC-1 (ATCC), RMA, and C4.4.25^-^, the β_2_m-deficient variant of the EL-4 lymphoma, were chosen as target cell lines. YAC-1 and RMA were cultured in suspension with RPMI1640 medium supplemented with 10% FBS, 1% Pen/Strep, 1 mM HEPES, while C4.4.25^-^ was cultured in DMEM medium supplemented with 10% FBS, 1% Pen/Strep, 1 mM HEPES.

### Flow Cytometry

The cells were washed twice with PBS buffer containing 1% BSA (Miltenyi) (FACS buffer) and were proceeded for surface staining by Fc block anti-mouse CD16/CD32 followed by fluorochrome-conjugated monoclonal antibodies, incubated at 4°C for 30 minutes, in the dark. Cells were then washed twice (100 μl, 4°C, 300 x g, 10 minutes, FACS buffer), and fixed with Cytofix/Cytoperm buffer (BD) for 45 minutes. Intracellular staining was performed after fixation/permeabilization, and staining was done in Perm/Wash buffer (BD) at 4°C for 30 minutes, in the dark. The samples were washed again and re-suspended in FACS buffer for further analysis. The acquisition was done on BD LSR Fortessa™. The monoclonal antibodies (mAbs) used for phenotypic and functional analyses are listed in **Table 1**.

**Table 1:** List of antibodies used.

Antibody to	Clone	Format	Supplier	Identifier
NK1.1	PK136	BUV-395	BD Biosciences	564144
		BV421	Biolegend	108741
CD3	145-2C11	BV510	BD Biosciences	563024
		PE-Cyanine7	eBioscience™	25-0031-82
NKG2A^B6^	16a11	PerCP-eFluor 710	eBioscience™	46-5897-82
Ly49C and Ly49I	5E6	BV786	BD Biosciences	744032
Ly49I	YLI-90	FITC	Thermo Fisher Scientific	A15413
KLRG1	2F1	BV421	BD Biosciences	562897
Qa-1^b^	6A8.6F10.1A6	BV421	BD Biosciences	744385
CD335 (NKp46)	29A1.4	BV421	BD Biosciences	562850
Qa-2	69H1-9-9	FITC	eBioscience™	11-5996-82
Ly49D	4 E5	FITC	BD Biosciences	555313
Ly49G2	4D11	FITC	BD Biosciences	555315
Ly49H	3D10	FITC	BD Biosciences	562536
H-2K^b^	AF6-88.5	PE	Biolegend	116507
Ly49F	HBF-719	PE	BD Biosciences	550987
CD314 (NKG2D)	CX5	PE	Biolegend	130207
CD19	1D3	PE/Cyanine7	BD Biosciences	552854
IFNγ	XMG1.2	BUV737	BD Biosciences	564693
TNFα	MP6-XT22	FITC	eBioscience™	11-7321-82
CD107a	1D4B	BV711	BD Biosciences	564348
Fixable Viability Stain 780			BD Biosciences	565388
CD16/CD32 (Mouse BD Fc Block™)	2.4G2	unconjugated	BD Biosciences	553142

### Degranulation Assay

NK cell degranulation towards target cells was tested by co-culturing them together for four hours at 37°C, in the presence of GolgiStop protein transport inhibitor (BD). Target cell lines YAC-1, RMA and C4.4.25^-^ were stained with CellTrace Violet at 0.5 mM concentration. The NK cells used were cultured in IL-2 and stimulated with IL-12/IL-15 and IL-12/IL-18 overnight. The Effector : Target (E:T) ratios were 2:1 and 5:1. The cells were then stained with mAbs to NK1.1, CD3, Ly49C/I, Ly49I, NKG2A, intracellular IFNγ and the fixable viability dye according to the staining protocol. The acquisition was done on BD LSR Fortessa™.

### Cytotoxicity

NK cell cytotoxicity towards target cells was tested by co-culturing them for four hours at 37°C. The NK cells used were cultured in IL-2 for six days and stimulated with IL-12/IL-15 and IL-12/IL-18 overnight before co-culture. The tested E:T ratios were 1:1, 5:1, 10:1, 25:1, 50:1. Target cell lines YAC-1, RMA and C4.4.25^-^ were stained with CellTrace Violet at 0.5 mM concentration. At the end of the four hours, TO-PRO-3 (1 µM, Invitrogen) diluted at 1:1000 was added to each sample. The aliquots were incubated at room temperature for 15 minutes and acquired on a NovoCyte Quanteon instrument.

### Statistics

Statistical analyses were performed in GraphPad Prism Version 9.3.1 for Windows (GraphPad Software, San Diego, CA, USA). Data points were plotted in grouped tables and the tests used to determine the statistical differences were One-way ANOVA (Tukey’s multiple comparisons test) or Two-way ANOVA (Dunnett’s or Sidak’s multiple comparisons tests), depending on the number of animals and parameters in the assay. We used p < 0.05 as the limit for statistical significance.

## Results

### Comparative Phenotype of B6 and TAP1-KO NK Cells

**Figure 1:**
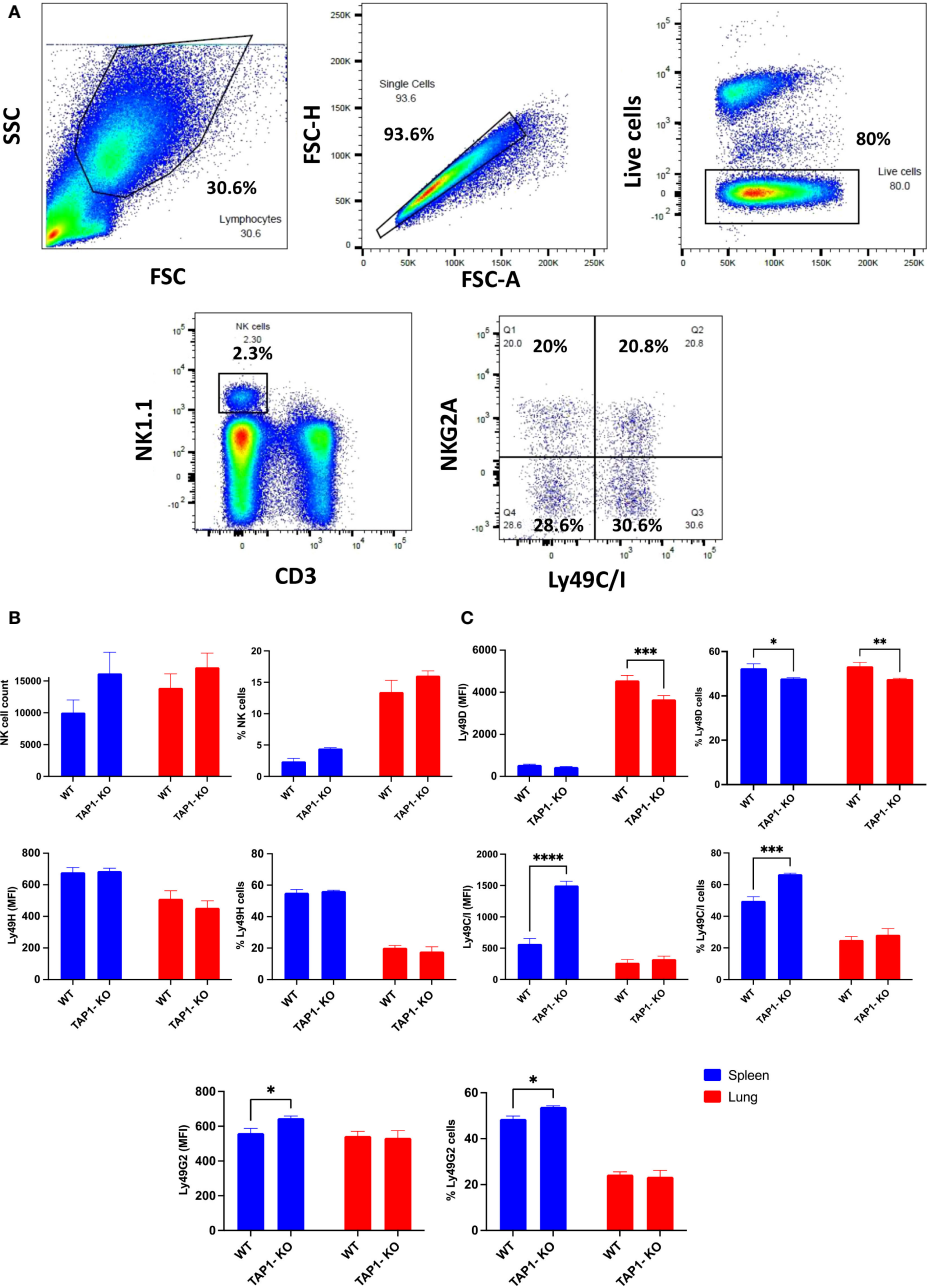
Comparison of NK cells from B6 and TAP1-KO littermates in spleen and in lung. **(A)** Gating strategy used to identify murine NK cells and their subsets. Representative flow cytometry plots of NK cells derived from mouse lung/spleen gated using the side and forward scatter dot plot display. NK cells were defined as CD3-NK1.1+ events and were further divided into NK cell subsets based on the expression levels of NKG2A and Ly49C/I. **(B)** Comparison of NK cell absolute count and frequency in live cells between wildtype B6 and TAP1-KO littermates derived from lung and spleen. **(C)** The comparison of some activating (Ly49D, Ly49H) and inhibitory (Ly49C/I, Ly49G2) members of the Ly49 family in spleen and in lung. Statistical analyses were performed using GraphPad Prism 9.0.0 with an ordinary two-way ANOVA with Sidak multiple comparisons tests (n=3). *, p<0.05, **, p<0.01, ***, p<0.001 and ****, p<0.0001.

We previously described the phenotype of B6 wildtype NK cells from spleen and lung, which displays several significant differences between the two organs. Notably, lung NK cells are more mature than their splenic counterparts (32). These findings, which were confirmed by others (33), have, to the best of our knowledge, not yet been investigated for the TAP1-KO strain. Splenocytes and lung mononuclear cells were stained with fluorescent antibodies (**Table 1**) and NK cells comparatively analyzed by flow cytometry. **Figure 1A** represents the gating strategy.

The proportion of NK cells (percentage of living cells that were CD3-NK1.1+) was almost threefold higher in lung than in spleen, whereas the absolute numbers were comparable (**Figure 1B**).

**Figure 2:**
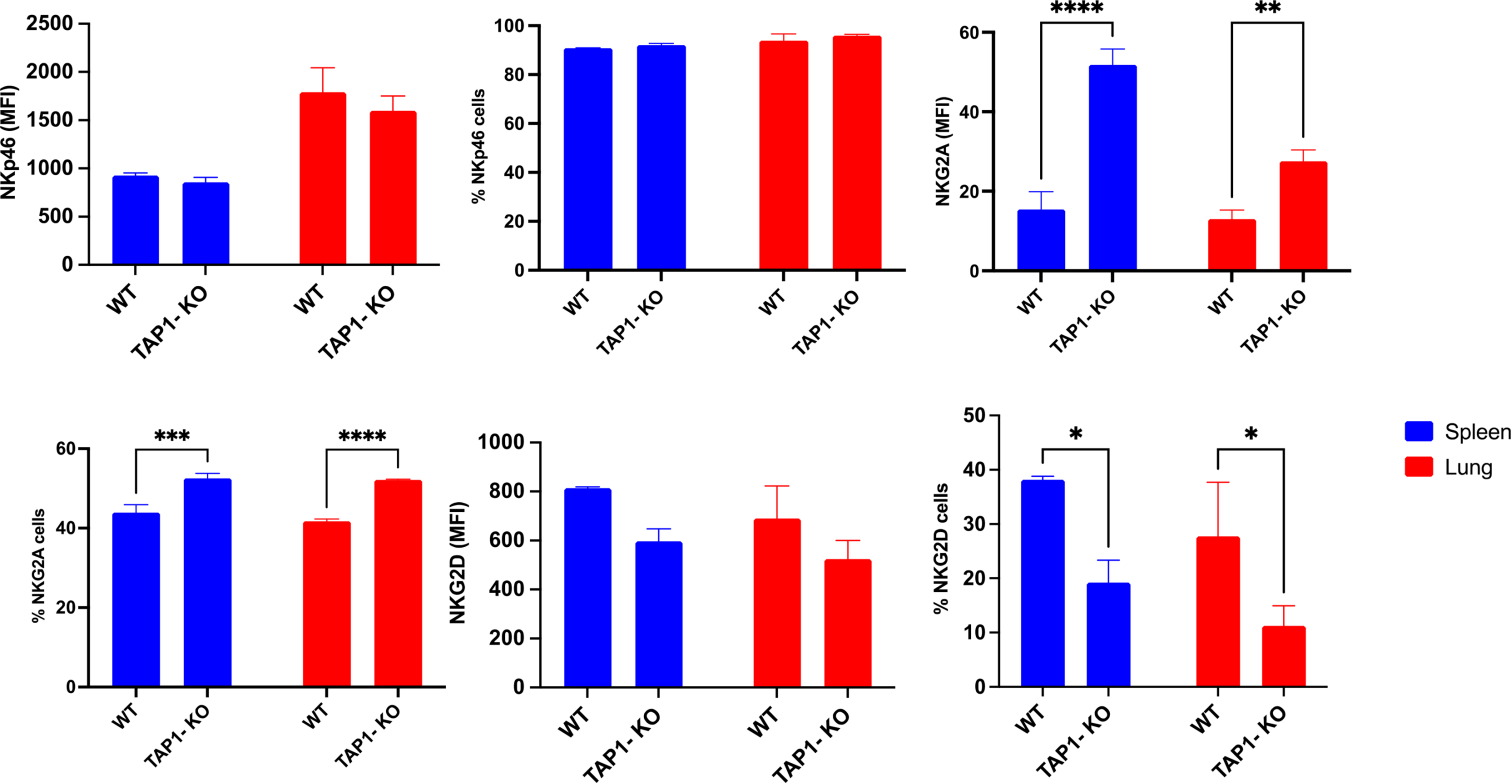
Comparison between expression levels of the natural cytotoxicity receptor NKp46 (AR), the C-type lectin NKG2D (AR) and the IR NKG2A. Statistical analysis was performed using GraphPad Prism 9.0.0 with an ordinary two-way ANOVA with Sidak multiple comparisons tests (n=3). *, p<0.05, **, p<0.01, ***, p<0.001 and ****, p<0.0001.

As described earlier for MHC class I-deficient mouse strains (30, 34), the splenic percentages (and MFI) of NK cells expressing Ly49C/I (**Figure 1C**) and NKG2A (**Figure 2**) were significantly higher in the TAP1-KO background than in the wildtype littermates.

In the lung, as previously observed (32) in B6 animals, the Ly49 IR were down- and the AR also down (Ly49H)-regulated or unchanged (Ly49D) (**Figure 1C**), with NKG2A remaining stable, compared to the spleen (**Figure 2**). For the TAP1-KO NK cells, the IR, including NKG2A (**Figures 1C**, **2**), still displayed an increased frequency of positive cells relative to the B6 wildtype mice, whereas the AR Ly49D and Ly49H were non significantly down-modulated.

We then turned to the analysis of the comparative expression between mice of the AR NKp46 and NKG2D (**Figure 2**), which are crucial for the cytotoxic activity of NK cells. The natural cytotoxicity receptor (NCR) NKp46 was present on almost all NK cells in the two types of mice and in spleen as well as in lung, with very similar expression levels per cell (reflected by the MFI), but overall higher MFI in lung than in spleen. Furthermore, the C-type lectin-type AR NKG2D showed a trend for lower frequencies and MFI in spleen and lung (no statistical significance) of TAP1-KO NK cells. However, the overall percentage of NKG2D+ NK cells was rather low compared to previous studies (35), which might be related to the antibody clone used.

**Figure 3:**
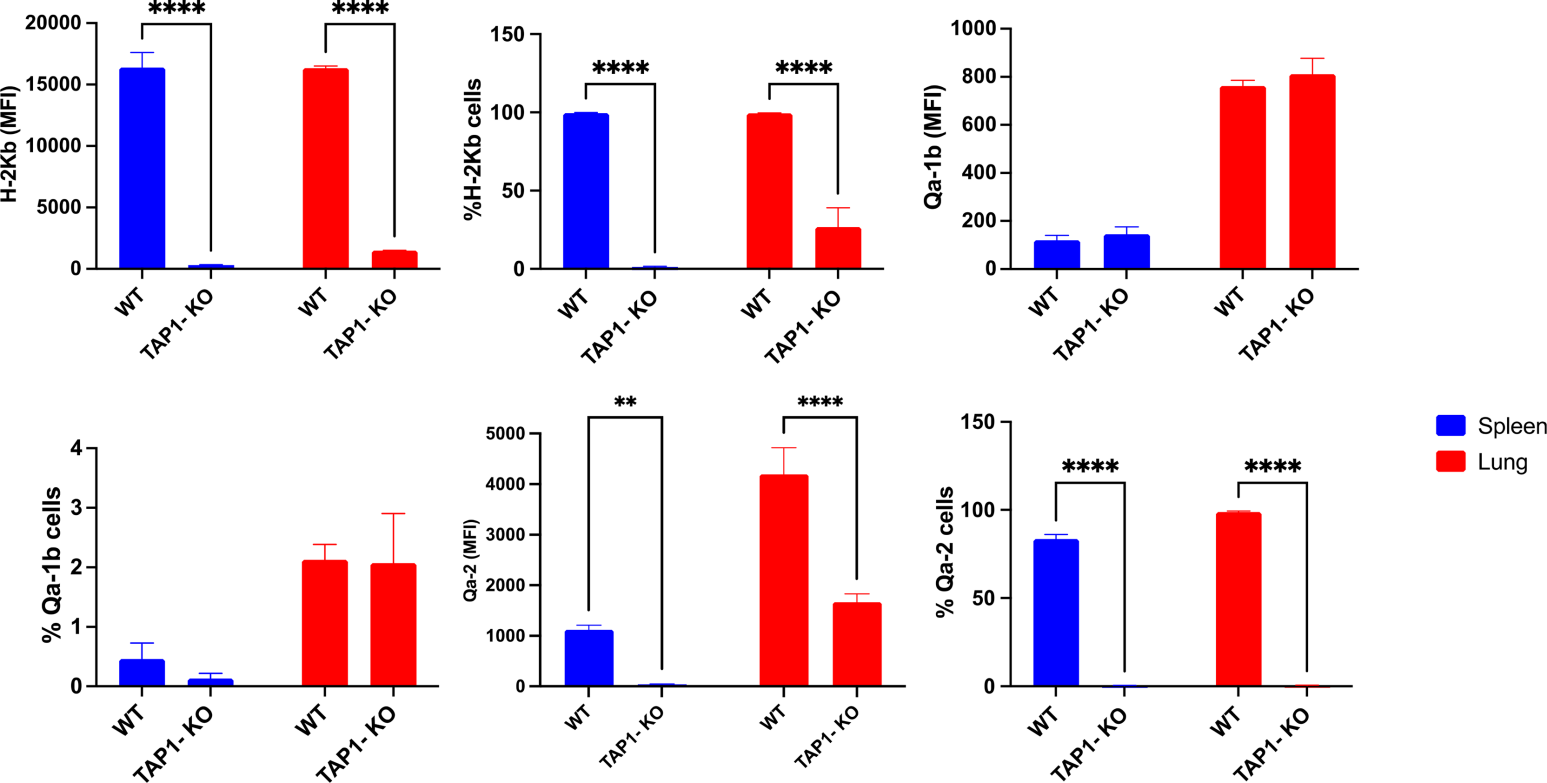
Expression and frequency of NK cell subsets based on MHC class I molecules H-2K^b^, Qa-2 and Qa-1^b^ in B6 wildtype and TAP1-KO mice. The NK cells were derived from lung and spleen, and stained and acquired by flow cytometry without expansion (*ex vivo*). Statistical analysis was performed using GraphPad Prism 9.0.0 with an ordinary two-way ANOVA with Sidak multiple comparisons tests (n=3). **, p<0.01 and ****, p<0.0001.

Although, as previously described (36), the classical MHC class I molecule H-2K^b^ was severely down-modulated but not absent from TAP1-KO cells (**Figure 3**), the non-polymorphic MHC class I molecule Qa-2 could not at all be revealed, whereas it was present on the totality of the NK cells from the B6 littermates (with, interestingly, a higher MFI in lung than in spleen). Thus, it can be concluded that this molecule, considered as the mouse equivalent of the human non-classical class I protein HLA-G (37), is entirely TAP-dependent and cannot be loaded with TAP-independent peptides, in contrast to Qa-1^b^ (38).

**Figure 4:**
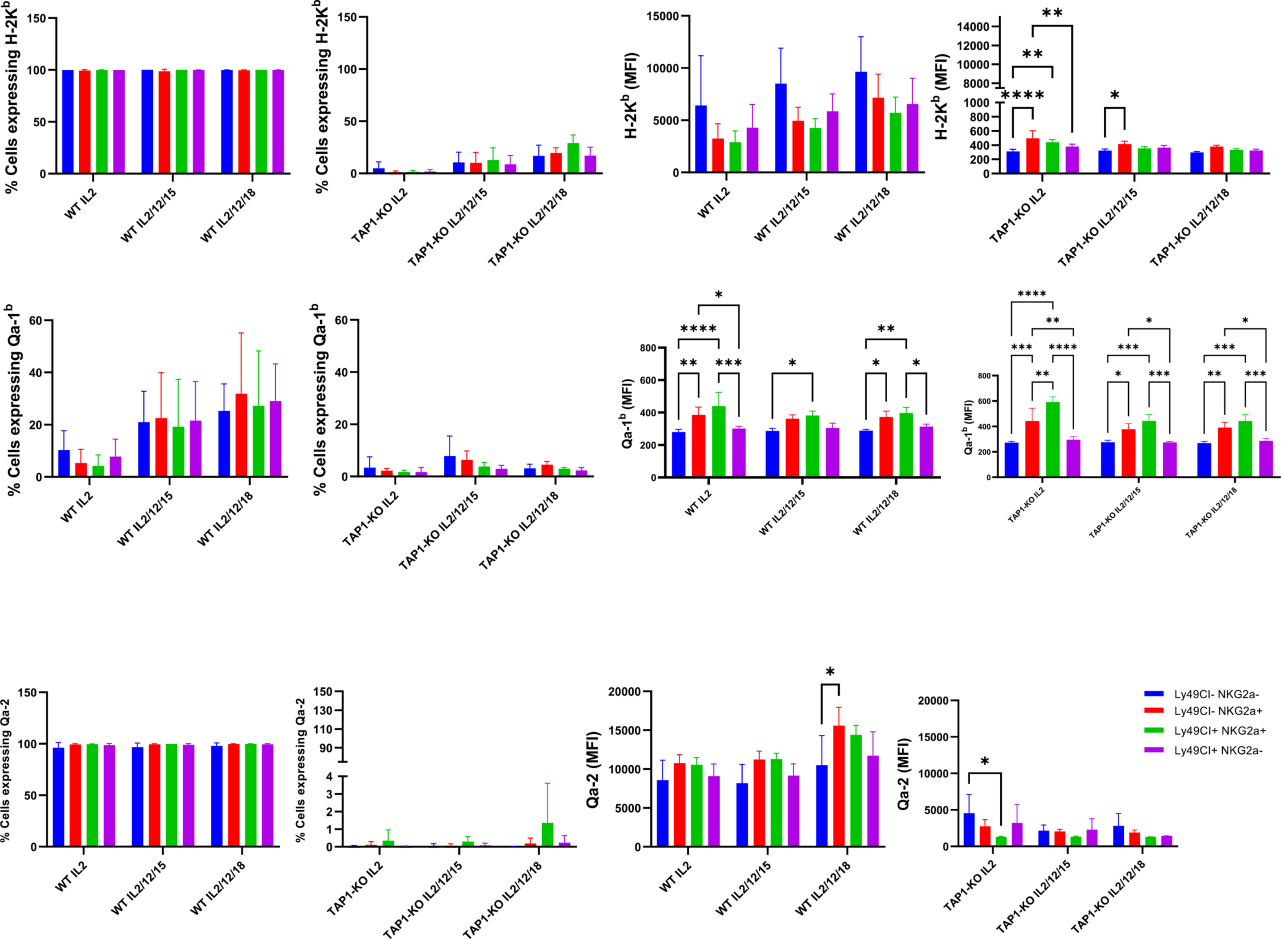
Comparison of MHC class I molecules H-2K^b^, Qa-2 and Qa-1^b^ in NK cells derived from spleens of the B6 and TAP-KO strains. The NK cells were expanded for 18 hours in the presence of IL-2 and stimulated with (IL-2, IL-12, IL-15) or (IL-2, IL-12, IL-18) with an IL-2 only control group as well. The expression levels were checked in the NK cell subsets defined by the presence or absence of NKG2A and Ly49C/I. Statistical analysis was performed using GraphPad Prism 9.0.0 with an ordinary two-way ANOVA with Tukey’s multiple comparisons tests (n=3). *, p<0.05, **, p<0.01, ***, p<0.001 and ****, p<0.0001.

We made interesting observations by comparing the expression levels of the MHC class I molecules H-2K^b^, Qa-2 and Qa-1^b^ in NK cells of the B6 and TAP-KO strains, after 18 hours of culture of *ex vivo* splenocytes in the presence of either IL-2 alone, (IL-2, IL-12, IL-15), or (IL-2, IL-12, IL-18) (**Figure 4**). The hypothesis here was that maybe these self-molecules would be differentially expressed by the four subsets defined by the presence or absence of Ly49C/I and NKG2A. As expected, the former two proteins (H-2K^b^ and Qa-2) were expressed homogeneously and at high levels in B6 mice, without major differences in the MFI between the subpopulations. However, we observed a double peak for H-2K^b^ in the Ly49C/I-NKG2A+ and Ly49C/I+NKG2A+ NK cells only in the (IL-2, IL-12, IL-18 condition). Our hypothesis held true in the case of Qa-1^b^ in the B6 strain. Indeed, whereas we found a homogeneous peak almost superposed to the negative control in the NK cells devoid of NKG2A, the two NKG2A+ subsets displayed a MFI overall approximately twice as high and moreover two connected but separate populations (one “bright” and one “dim”) in the three cytokine conditions. The significance of this data will have to be analyzed in further experiments.

Furthermore, while the expression of H-2K^b^ was very low in TAP1-KO NK cells, again as expected, the effect of IL-12 + IL-18 was still observable even in this MHC class I-deficient context (**Figure 4**). Very interestingly, for Qa-1^b^, the same phenomenon of the double populations appeared selectively in the NKG2A+ NK cells of the three mixtures of cytokines. This suggests both in B6 wildtype and in TAP1-KO NK cells, that the observation is linked to NKG2A rather than to the types of cytokines and/or the MHC class I molecules themselves.

**Figure 5:**
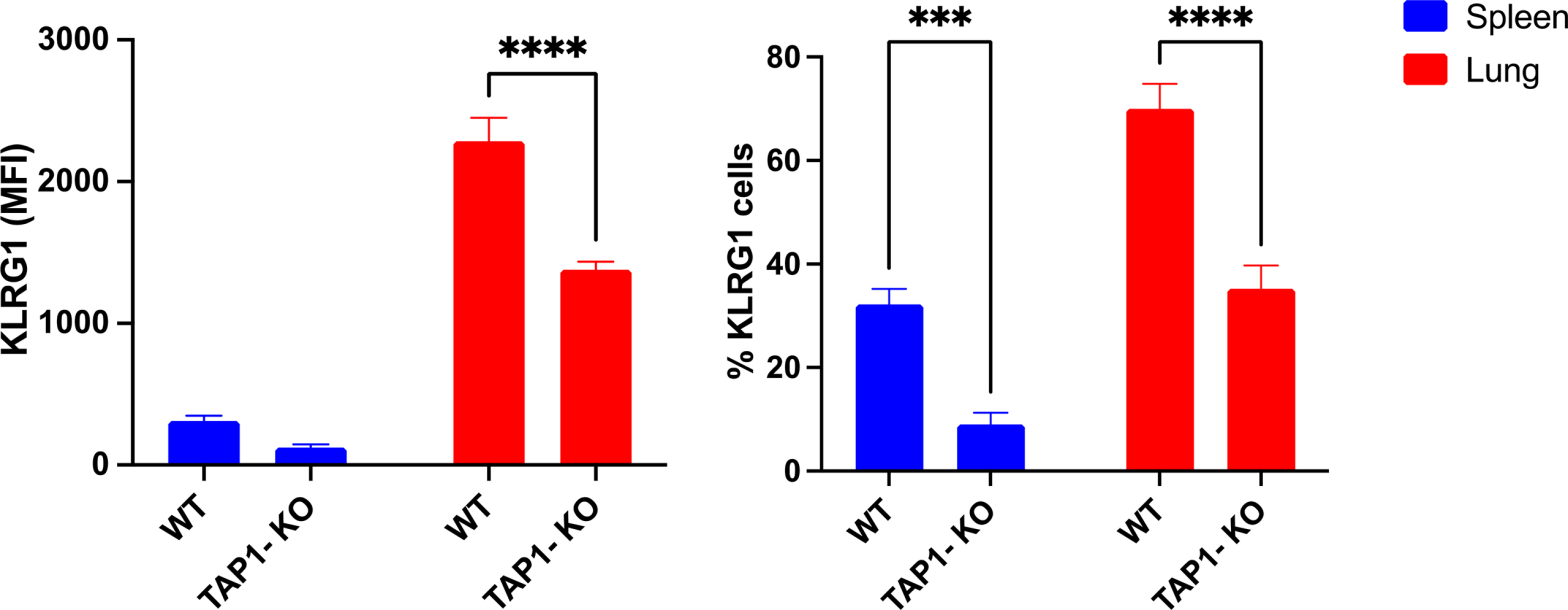
KLRG1 expression levels and frequency measured in murine lung- and spleen-derived NK cells. Statistical analyses were performed using GraphPad Prism 9.0.0 with an ordinary two-way ANOVA with Tukey’s multiple comparisons tests (n=3). ***, p<0.001 and ****, p<0.0001.

We concluded our phenotypic investigations with the senescence and activation marker KLRG1 (**Figure 5**), better represented on terminally differentiated NK lymphocytes (39). KLRG1 was expressed on a significantly reduced NK cell population of β2m-KO mice of various genetic backgrounds (40), an observation that we could confirm in the spleens of TAP1-KO animals, both in terms of frequency of KLRG1+ cells and of MFI. Interestingly, the percentages of the KLRG1+ NK cells and the MFI of the receptor showed a strong trend to increase in both types of mice in the lung compared to the spleen, although TAP1-KO NK cells still lagged behind wildtype for both of the parameters. Overall, the data shows that NK cells from TAP1-KO mice are relatively less senescent and activated compared to their wildtype counterparts.

### Differences in Activated NK Cell IFNγ Production According to the Initial Licensing Status

**Figure 6:**
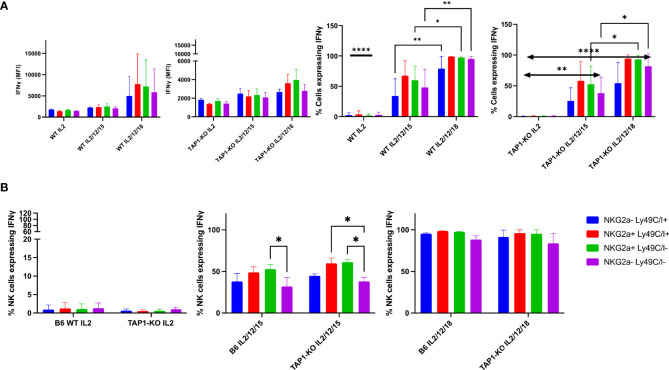
**(A)** IL-2 expanded B6 WT and TAP1-KO NK cells from murine spleens were activated with overnight stimulation of cytokines IL-2, (IL-2, IL-12, IL-15) and (IL-2, IL-12, IL-18). At day 1, IFNγ was measured in CD3-NK1.1+ NK cells by intracellular flow cytometry staining. Statistical analysis was performed using GraphPad Prism 9.0.0 with an ordinary two-way ANOVA with Sidak multiple comparisons tests (n=3). *, p<0.05, **, p<0.01 and ****, p<0.0001. **(B)** B6 WT and TAP1-KO NK cells from murine spleens were expanded in the presence of IL-2 for 5 days. On day 5, NK cells were activated with overnight stimulation of cytokines IL-2, (IL-2, IL-12, IL-15) and (IL-2, IL-12, IL-18). IFNγ was measured in CD3-NK1.1+ NK cells by intracellular flow cytometry staining. Statistical analysis was performed using GraphPad Prism 9.0.0 with an ordinary two-way ANOVA with Sidak multiple comparisons tests (n=3). *, p<0.05, **, p<0.01 and ****, p<0.0001.

Next, we wanted to compare the production of the signature cytokine of NK cells, IFNγ, in the four subsets defined by the presence or absence of the self-specific and educating IR, Ly49C/I and NKG2A. Theoretically, as outlined above, we expected fewer IFNγ-producing NK cells in the double negative, unlicensed NK cell fraction, as described by Kim et al. (10). This dataset was nevertheless established in *ex vivo* splenocytes stimulated with plate-bound antibodies. We asked the question if the licensing features would be maintained after a strong stimulation with the cytokines IL-2, (IL-2, IL-12, IL-15) and (IL-2, IL-12, IL-18), and investigated this initially at day 1, *i.e.* after an overnight activation (**Figure 6A**). Here, we found in B6 wildtype NK cells no significant IFNγ production with IL-2 alone, whereas all four subsets defined by Ly49C/I and NKG2A produced the cytokine with significant values around or above 50% for the two NKG2A+ subpopulations *versus* approximately 30% to 40% for the NKG2A- ones when stimulated by the (IL-2, IL-12, IL-15) cocktail. Ly49C/I+ NK cells contained more IFNγ-producers than the double negative subset. In the presence of (IL-2, IL-12, IL-18), between 80% and 100% of NK cells produced the cytokine. This observation, at least regarding (IL-2, IL-12, IL-15), fits with current models for NK cell education, notably the licensing model (10) and the rheostat model (10–12, 23, 24). The latter predicts higher functional capability of NK cells when they express more than one self-specific IR. Natural killer cells from TAP1-KO mice were stimulated under the same conditions. Whereas again no IFNγ production was observed in the presence of IL-2 alone, the two cytokine cocktails surprisingly induced an almost similar distribution of the IFNγ-producing NK cells compared to wildtype mice, with the strongest effect seen in NKG2A+ cells, followed by Ly49C/I single positives and finally the double negatives (barely half the values of the NKG2A+ cells). In principle, as TAP1-KO NK cells cannot be appropriately educated, one should not observe such a difference in IFNγ-producing cells between the NK subsets but, as even MHC class I- NK cells become activated under appropriate cytokine stimulation (18), a more equal distribution.

To get a deeper insight into this topic, we repeated the experiments by first culturing splenocytes with IL-2 alone for five days and then, at day 5, adding the same three cytokine combinations until day 6, when the cells were harvested and stained (**Figure 6B**). We then similarly looked at the percentages of IFNγ+ NK cells in the four subsets defined by the presence or absence of the IR Ly49C/I and NKG2A.

Overall, we observed the same results as at day 1, although the global cytokine production levels were higher in each subset. The double negative population was still the less proficient in intracellular IFNγ accumulation in the (IL-2, IL-12, IL-15) condition, whereas the NKG2A+ subsets remained the most productive ones. With (IL-2, IL-12, IL-18), almost 100% of the four subpopulations became IFNγ+. However, concordant differences were still observed in the MFI, reflecting in these cases the quantity of IFNγ per cell and not, as the percentages parameter, the fraction of NK cells producing the cytokine. Similar to day 1, we could not observe significant differences between B6 wildtype and TAP1-KO mice, meaning that (i) the wildtype NK cells maintained to some degree the *ex vivo* educational profile that left an imprint even after a very strong cytokine stimulation at days 1 and 5, and (ii) this imprint was also present in the TAP1-KO context, although NK cells are not supposed to be educated through classical and non-classical MHC class I molecules in this genetic background (as the expression of these proteins is severely reduced). Moreover, our data validate the licensing (10) and the rheostat models (10–12, 23, 24) even in a situation of major NK cell activation.

**Figure 7:**
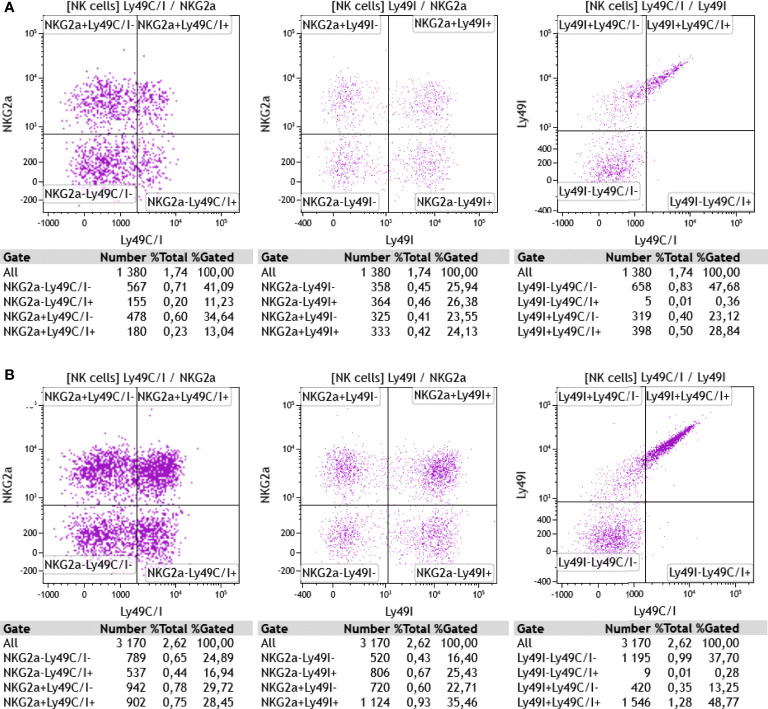
**(A)** *Ex vivo* staining of CD3-NK1.1+ splenocytes from a wildtype B6 mouse with anti-NKG2A^B6^ (clone16a11), anti-Ly49C/I (clone 5E6), and anti-Ly49I (clone YLI-90) monoclonal antibodies. **(B)** Same antibodies used for the staining of splenocytes from a TAP1-KO littermate. One representative experiment out of two is shown (n=2). Particularly in the wildtype cells, the discrimination of Ly49C/I+ NK cells from the Ly49C/I- population is suboptimal, which might be explained, at least in part, by a competitive effect of the YLI-90 antibody for epitopes shared with 5E6. In the TAP1-KO mouse, the MFI of Ly49I on NK cells expressing this receptor is higher than in the B6 wildtype, suggesting that Ly49I, similar to Ly49A and Ly49C for example, tries to adapt to the missing MHC class I molecules through up-regulation.

Given the previously mentioned fact that the 5E6 antibody recognizes both Ly49C and Ly49I, but that in the B6 wildtype animals, Ly49C might be masked due to its *cis* interaction with H-2K^b^, we also stained the cells with the Ly49I-specific antibody YLI-90. Here, we found at day 0 (*ex vivo*) nicely separated subpopulations of Ly49I+/- and/or NKG2A+/- NK cells (**Figure 7**) in both wildtype and TAP1-KO mice, the latter displaying a highly increased MFI (155% relative to B6 wildtype) for Ly49I. In contrast, the Ly49C/I versus Ly49I staining revealed a double positive diagonal population, as observed in flow cytometry by staining the same molecules or closely related epitopes. Finally, the percentages of Ly49C/I+ cells *versus* NKG2A+ events in the wildtype mice were abnormally low, which could reflect steric hindrance between 5E6 and YLI-90.

**Figure 8:**
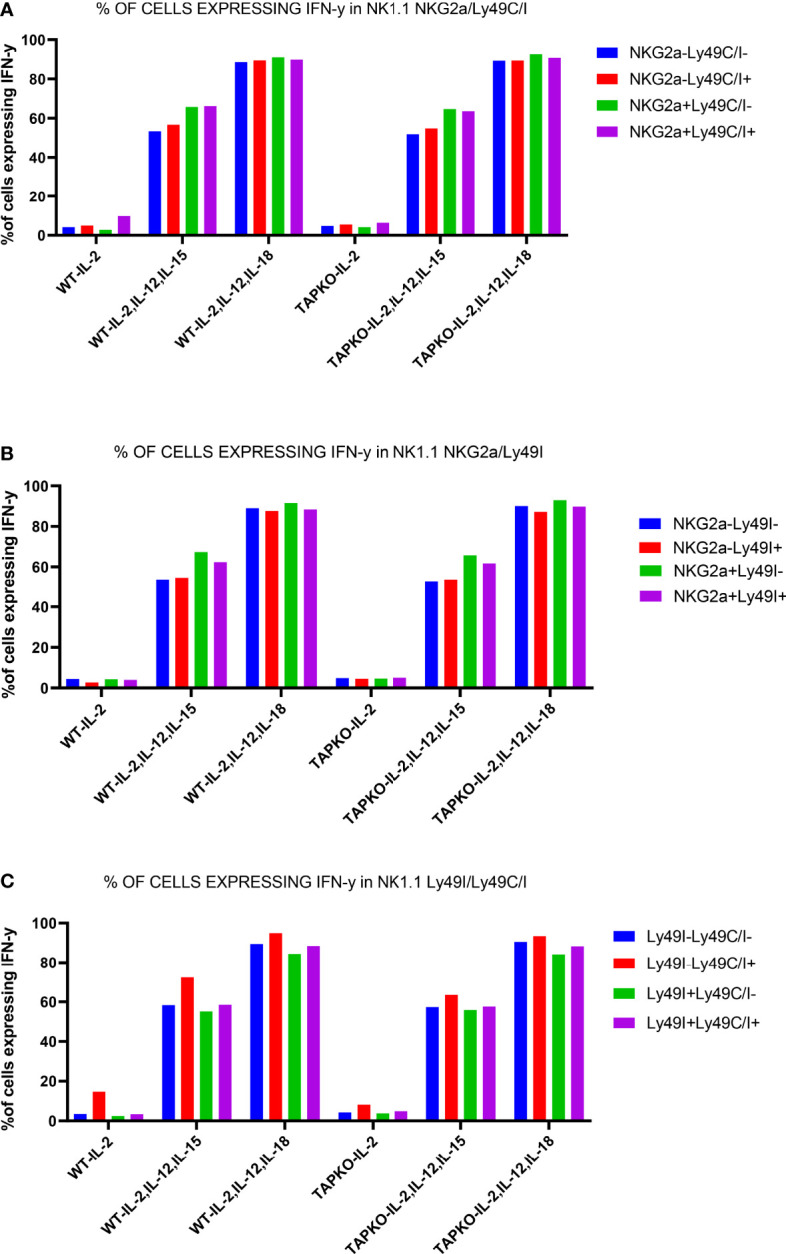
One representative experiment out of two showing the educational impact of Ly49C, but not of Ly49I, at day 6 in cytokine-stimulated NK cells. **(A)** Comparison of IFNγ production in CD3-NK1.1+ splenocytes activated with IL-2, (IL-2, IL-12, IL-15) and (IL-2, IL-12, IL-18) among the subsets defined by the presence or absence of Ly49C/I and NKG2A. **(B)** The same readout for NK cells expressing or not the IR Ly49I and NKG2A. **(C)** Comparison of the differences in IFNγ production in the NK cell subsets defined by the presence or absence of Ly49C/I and Ly49I. The higher production in the Ly49C/I+ population suggests an educational impact of Ly49C under our activation conditions, but no or only a very weak influence of Ly49I.

We then repeated the IFNγ-producing experiments at day 6 and measured the level of the cytokine in the Ly49C/I, Ly49I and NKG2A NK cell subsets compared with the receptor-negative counterparts. Overall, for Ly49C/I and NKG2A, the data shown in **Figure 6B** were confirmed (**Figure 8**). Interestingly, Ly49I did not seem to contribute to NK cell licensing, as the Ly49I+ subset did not produce more IFNγ than the Ly49I- populations. In contrast, the Ly49C/I+ NK cells harbored a distinctly higher percentage of cytokine-producing NK cells than their counterparts negative for IR. Thus, as this increase cannot stem from Ly49I, we consider our results as evidence for the educational imprint of Ly49C.

### Downmodulation of Ly49C/I and Ly49I on Activated NK Cells at Day 6

**Figure 9:**
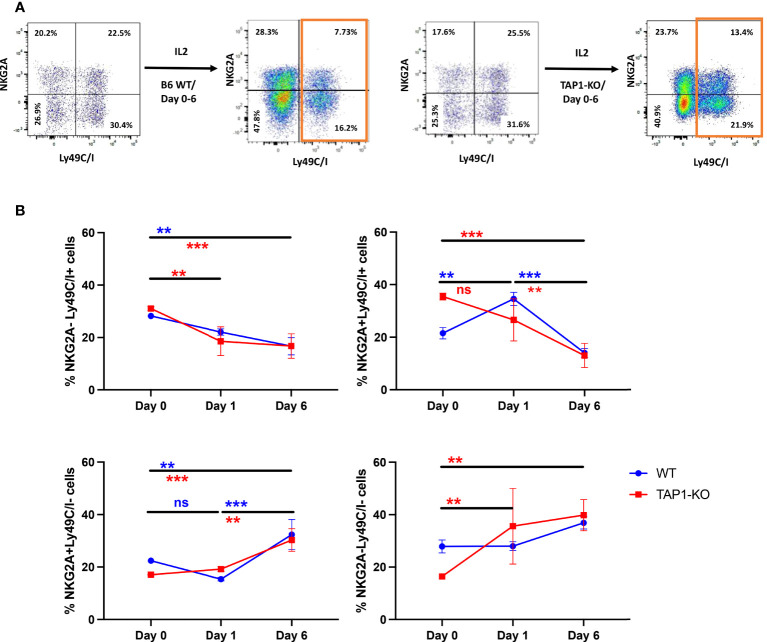
**(A)** Representative flow cytometric dot plots of splenic NK cells distinguished according to NKG2A and Ly49C/I expression. The NK cells were expanded in the presence of IL-2 for 6 days and the expression levels of NKG2A and Ly49C/I were compared with that of day 0. **(B)** Analyses of the frequency of NK cell subsets based on NKG2A and Ly49C/I expression levels. The frequency between day 0, day 1 and day 6 for IL-2 expanded NK cells was compared. Statistical analysis was performed using GraphPad Prism 9.0.0 with an ordinary two-way ANOVA with Sidak multiple comparisons tests (n=3). **, p<0.01, ***, p<0.001 and ns, not significant. The colors of the * correspond to the strains of mice used.

When we undertook the phenotyping of the spleens at day 6 for the purpose of measuring intracellular IFNγ production by NK cells, we observed that the percentages of Ly49C/I+ events were dramatically down-modulated compared with days 0 (*ex vivo*) and 1. This phenomenon (also noticed in the case of Ly49I but not of NKG2A) was significant for each mouse type, either between day 0 and day 6 (NKG2A-Ly49C/I+) or between day 1 and day 6 (double positive), whereas rather minor and not systematically significant differences could be seen between day 0 and day 1 (**Figure 9**). In a mirror image, the percentages of NKG2A+Ly49C/I- as well as double negative NK cells increased. A reduction of Ly49C/I+ NK cells in the context of activated tumor-infiltrating lymphocytes has been previously described by Shi et al. (41).

### Natural Killer Cell Cytotoxic Activity

Besides cytokine production, natural cytotoxicity against tumor cells and viral-infected cells is the second major property of NK cells. In the mouse, the lymphoma cell line YAC-1 is the standard target, due to its exquisite NK cell sensitivity (42). Another T cell lymphoma, RMA, is rather resistant to these cells, whereas C4.4-25^-^, a β2m-deficient variant of EL4, the parental cell line of RMA (43), is again susceptible because of the almost absent expression of MHC class I molecules (44). As the latter two possess the B6 genetic background, they can serve for the determination of the missing self-recognition by NK cells from B6 origin.

**Figure 10:**
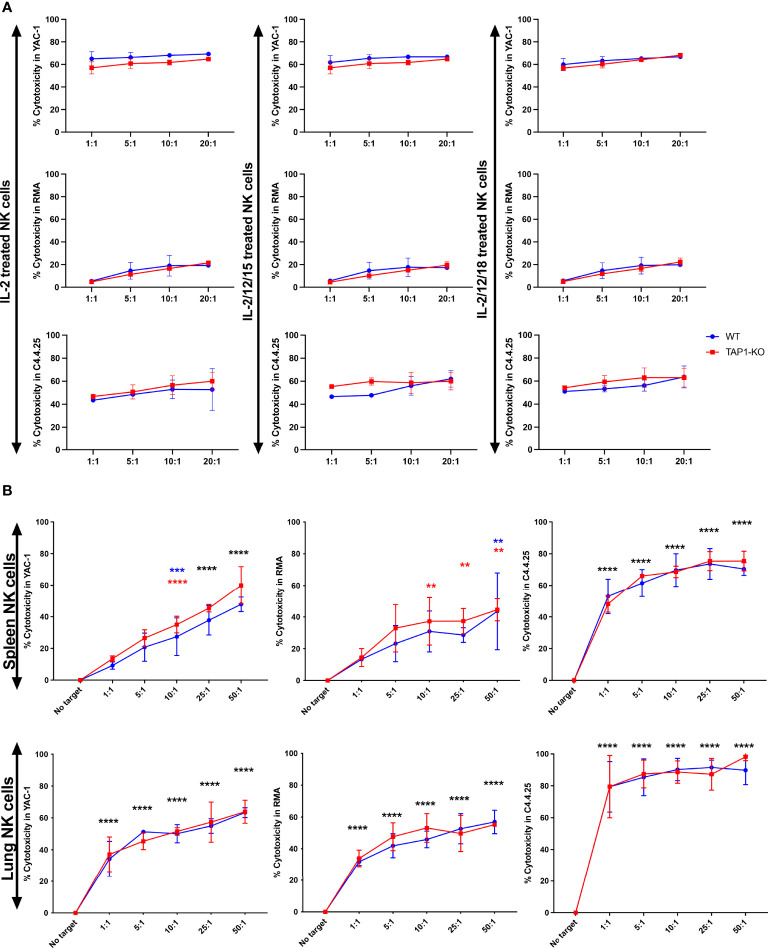
**(A)** NK cells derived from murine spleen were expanded overnight in IL-2 and stimulated with IL-2 alone, (IL-2, IL-12, IL-15) or (IL-2, IL-12, IL-18) cytokines overnight. These cells were then co-cultured with the targets YAC-1, RMA and C4.4-25- for four hours. The cytotoxicity was measured with TO-PRO-3 staining. **(B)** NK cells derived from murine spleen and lung were expanded in the presence of IL-2 for 5 days and then restimulated with IL-2 alone overnight. These cells were then co-cultured with the targets YAC-1, RMA and C4.4-25- for four hours. The cytotoxicity was measured with TO-PRO-3 staining. **, p<0.01, ***, p<0.001, ****, p<0.0001.

We performed a four-hour cytotoxicity assay in duplicates, with splenocytes from the two types of mice cultured overnight with IL-2 alone and with each of the two cytokine cocktails and evaluated the lysis of the targets YAC-1, RMA and C4.4-25^-^ (**Figure 10A**). It is known that a relatively short incubation time with IL-2 is enough to significantly increase natural cytotoxicity (45), and this was the case in both strains, as YAC-1 was killed at levels between 50% and nearly 70% at all E/T ratios. Interestingly, the addition of the other cytokines did not further level up the cytotoxicity, but even induced a minimal trend towards a decrease.

RMA cells resisted quite well as described (44) and the residual level of cytotoxic activity, that became significant at the highest E/T ratios, was again observed to a comparable degree between the two types of splenocytes. Furthermore, we demonstrated that B6 wildtype and TAP1-KO NK cells were able to perform missing self-recognition, as they abundantly killed the MHC class I-deficient cell line C4.4-25^-^, which lacks the B6 class I molecules H-2D^b^ and H-2K^b^, highly expressed by RMA (data not shown). Thus, after cytokine stimulation, NK cells from TAP1-KO become functional to the same extent as their wildtype counterparts.

Then, we repeated the cytotoxicity experiments with spleen and lung NK cells cultured during five days in the presence of IL-2 and re-stimulated overnight with IL-2 alone (**Figure 10B**). Here again, YAC-1 and C4.4-25^-^ were very efficiently lysed by NK cells from the two strains and of both organs. Lung NK cells showed a tendency to a stronger killing activity than their splenic counterparts. RMA cells were significantly susceptible to spleen and lung NK cells (especially at higher E/T ratios), which might be related to the longer stimulation time of these effectors compared with only one day. Importantly, we could not detect statistically significant differences in the lysis intensity between B6 and TAP1-KO NK cells, confirming the results obtained at day 1 with the spleen and extending them at day 6 to the lung.

### Natural Killer Cell Degranulation

**Figure 11:**
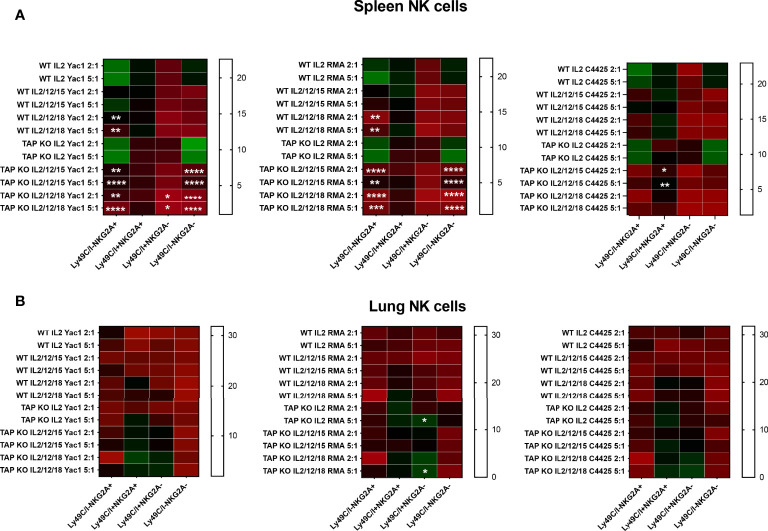
**(A)** NK cells derived from murine spleen were expanded in the presence of IL-2 for five days and then restimulated with IL-2 alone, or (IL-2, IL-12, IL-15) or (IL-2, IL-12, IL-18) cytokines overnight. These cells were then co-cultured with the targets YAC-1, RMA and C4.4-25- for four hours. The stimulated cells were investigated for CD107a expression among CD3-NK1.1+ NK cells by flow cytometry staining. The expression levels were divided into four NK cell subsets based on the presence or absence of NKG2A and Ly49C/I. Statistical analysis was performed using GraphPad Prism 9.0.0 with an ordinary two-way ANOVA with Tukey’s multiple comparisons tests (n=3). *, p<0.05, **, p<0.01, ***, p<0.001 and ****, p<0.0001. (B) Same experiments, but with NK cells derived from murine lungs.

Degranulation assays consist in the measurement, by flow cytometry, of the percentage of NK cells expressing the marker CD107a at the surface after, for example, incubation with target cells (46). This molecule is part of the membrane of the NK cytotoxic granules and accompanies their secretion. It is considered as a surrogate for the cytolytic activity (46). We analyzed degranulation of *ex vivo* spleen and lung NK cells from the two types of mice (three mice per strain) after overnight stimulation with the same cell lines and cytokine cocktails used for the cytotoxicity evaluation in the various quadrants defined by the presence or absence of Ly49C/I and NKG2A (**Figures 11A, B**). Quite surprisingly, there was a significant level of degranulation (CD107a+ NK cells) at baseline, *i.e.* in the absence of targets. This background was higher with IL-2 alone than with the two mixtures of interleukins, but predominantly among the NKG2A single positive and the double positive NK cells. The values did not change significantly after addition of the three target cells but continued to be highest with IL-2 alone. Furthermore, we observed the same distribution of the percentages of de-granulating cells than in the experiments about IFNγ production, namely that the most important fractions of CD107a+ NK cells were reached in the two subsets expressing NKG2A, compared to the Ly49C/I single positive and the double negative populations. Whereas IL-2 was most efficient in stimulating spontaneous and target cell-induced CD107a cell surface mobilization, it came a bit unexpected that the cytokine cocktails were less active here. In any case, the degranulation experiments confirmed once more published data about NK cell licensing and education.

## Discussion

In this study, we confirm the initial licensing profile of NK cells in the B6 wildtype mouse genetic background, corresponding to the fact that the expression of self-specific IR for autologous (self) MHC class I molecules educates these cells and renders them functionally active, although the licensing does not automatically lead to the activation of the entire population of self-specific IR+ NK cells but only of a fraction of them, in accordance with the literature (10, 11). The choice of TAP1-KO mice stems from the fact that their NK cells are usually considered as non-educated and hypo-reactive (36), in contrast to standard B6 mice, and are therefore an optimal model for a study about licensing.

However, the phenotype and functions of TAP1-KO NK cells are not that different from their wildtype counterparts, and in many knockout models of NK cell receptors or members of signaling cascades, the surface phenotype remains unaffected.

Our experiments furthermore confirmed the phenotypic differences between spleen and lung NK cells that we (32), and others (33), have previously shown. The latter appear as more mature at least in the mouse (32, 33). This is fully in line with the current paradigm of NK cell heterogeneity dependent on the organ that is looked at (47), and so the mere investigation of peripheral blood NK cells in human and/or splenic NK cells in the mouse, although they are easily accessible, is to some extent a limitation. This might have clinical consequences in human patients, if for example an immunotherapeutic modality targeting NK cells would have organ-specific effects that could be either favorable or detrimental depending on the specific population.

Interestingly, the phenotype of TAP1-KO NK cells was only minimally different from wildtype, as previously described (36). However we found that after a strong cytokine-mediated activation, these cells resembled wildtype NK cells in terms of licensing. Indeed, the level of autologous MHC class I molecules being very low in their cellular environment, they are hyporeactive *ex vivo* and can in principle not be educated. Nevertheless, after stimulation with (IL-2, IL-12, IL-15) or (IL-2, IL-12, IL-18), they become significantly active in terms of cytokine production and cytotoxicity. What was really surprising was the observation that, although the four NK cell subsets defined by the presence or absence of the self-specific IR were significantly active regarding IFNγ production, which was largely expected in the wildtype mice, the initial education profile was to some extent conserved at day 6, and moreover, also present and clearly identifiable in the TAP1-KO NK cells. Thus, there must be molecular processes that can license these cells independently of MHC class I molecules and on the other hand are likely related to them, as the differences are defined by the presence or absence of Ly49C/I and NKG2A.

The phenotypic analysis of the NK cells from the two strains revealed some interesting differences, which might be worth investigating in more detail and with more fluorochromes for flow cytometry and/or an investigation by mass cytometry. We were particularly impressed by the total absence of staining of TAP1-KO cells by the anti-Qa2 antibody, revealing the complete dependence of this non-classical HLA class I molecule on a functional TAP.

The downmodulation of Ly49C/I, whereas NKG2A expression was spared, has previously been described by Shi et al. (41). These authors envisaged several possibilities to explain the observation, namely receptor down-modulation (internalization?), contraction of the subset or relative expansion of the Ly49C/I- NK cells, to finally suggest after an experimental approach that the most likely explanation might be receptor shedding from the cell surface. In any case, the phenomenon could be reproduced after cytokine-mediated stimulation of NK cells as an incidental finding in our study. If the reason behind it was receptor internalization after prolonged contact with cognate MHC class I ligands in the cell culture system, it would most likely not have been observed in TAP1-KO mice. Comparative evaluation of the proliferation of Ly49C/I- compared with Ly49C/I+ NK cells, and the amount of soluble Ly49C/I in the supernatant of strongly activated *versus* less activated splenocyte cultures could be interesting perspectives for future experiments. Likewise, Korten et al. reported a reduction in the percentages of Ly49C+, Ly49G2+ and Ly49A+ NK cells in helminth infection, in the context of a global NK cell expansion (48). However, these observations stem from the Balb/c mouse strain, which carries another genetic background and other polymorphic forms of the Ly49 family. Tay et al. showed that in murine cytomegalovirus infection, the percentage of Ly49C+ NK cells declines in the spleen but not in the peritoneal exudate, a phenomenon that is not present in lymphocytic choriomeningitis virus (LCMV) infection (49). Other aspects of Ly49C/I receptors are their presence on memory NK cells and their peptide sensitivity (recognition of the peptide presented by the cognate MHC class I molecules), as reviewed by Wight et al. (50). One might hypothesize that the loss of Ly49C/I by activated NK cells renders these effectors more efficient against targets expressing classical MHC class I ligands. After cytokine-mediated stimulation however, the most efficient activation resides in the NKG2A-expressing subsets, which seem to educate NK cells “better” than Ly49C/I. Finally, Ly49C also interacts with its ligand in *cis*, *i.e.* in the plane of the same cell membrane (51, 52), which corresponds in fact to the physiological situation. This phenomenon, initially described for Ly49A, has important consequences for the education and function of NK cells (26).

Despite their biological immune suppression (low number of CD8+ T cells, hypo-reactive NK cells), TAP1-KO mice display no clinical phenotype, if they live in a specific pathogen free or a “dirty” animal facility. Moreover, their short life span and the normal humoral immune response, as well as the possibility of activating their NK cells in an infectious context, might contribute to the explanation. In addition and importantly, Barbet et al. recently described that a “non-canonical” way of cross-presentation by TAP-deficient dendritic cells allows the stimulation of CD8+ T cells (53). This is in sharp contrast to human TAP deficiency, a very rare autosomal recessive disease (around 40 cases described in the literature) characterized by repeated bacterial infections of the respiratory tract, bronchiectasis, deep skin ulcers and a granulomatous destruction of the nasal cartilage ending up in a clinical picture resembling NK/T cell lymphoma, nasal type (lethal midline granuloma) (54). These patients usually reach early adulthood but have a low quality of life. Their NK cells are hypo-reactive *ex vivo* as in the mouse, but they become strongly activated after cytokine stimulation (IL-2). They present aroused NK cells within the skin lesions, so that we cannot exclude that the former contributes to the pathophysiology of the disease (13, 14, 18). A mouse model mimicking skin ulcers and/or bacterial respiratory infections would be necessary to address these questions in-depth.

Overall, we confirm the licensing (an IR on the NK cell must interact with an autologous MHC class I molecule) and the rheostat (the more IR there are on a NK cell, the better it is educated – quantitative aspect) models that surprisingly also seem to apply to the TAP1-KO NK cells, whereas this is theoretically not possible. The only explanation would be that Qa-1^b^ might educate the cells, as its expression is possible with TAP-independent peptides (55). An argument for this possibility could be that the NKG2A+ NK cell subsets react stronger than the NKG2A- ones, at least in the case of the parameters we investigated. An interesting observation is the loss of Ly49C/I, that could indicate, if similar observations would be made for human KIR and/or NKG2A, the possibility of selecting subsets with lower percentages of IR+ NK cells. This would in turn optimize adoptive NK cell therapies, because the majority of the cells would be free from inhibition by the host’s MHC class I molecules and therefore, at least in theory, be more effective than a bulk population in eradicating tumors or infections.

## Data Availability Statement

The original contributions presented in the study are included in the article/supplementary material. Further inquiries can be directed to the corresponding author.

## Ethics Statement

The animal study was reviewed and approved by the Animal Welfare Structure of the Luxembourg Institute of Health and by the Ministry of Health, Luxembourg.

## Author Contributions

NP, CM, MauT, OS, CNAA and MarT performed experiments and analyzed data. CS-D and HS gave critical input to the study. MO and JZ conceived and supervised the work. NP and JZ wrote the paper. All authors edited the paper and agreed with the final version.

## Funding

Salary of the PhD student (NDP): Fonds National de la Recherche Luxembourg, FNR PRIDE/11012546/NEXTIMMUNE. Other funding: Ministry of Higher Education and Research, Luxembourg.

## Conflict of Interest

The authors declare that the research was conducted in the absence of any commercial or financial relationships that could be construed as a potential conflict of interest.

## Publisher’s Note

All claims expressed in this article are solely those of the authors and do not necessarily represent those of their affiliated organizations, or those of the publisher, the editors and the reviewers. Any product that may be evaluated in this article, or claim that may be made by its manufacturer, is not guaranteed or endorsed by the publisher.
